# High-Throughput Identification of Nuclear Envelope Protein Interactions in *Schizosaccharomyces pombe* Using an Arrayed Membrane Yeast-Two Hybrid Library

**DOI:** 10.1534/g3.120.401880

**Published:** 2020-10-27

**Authors:** Joseph M. Varberg, Jennifer M. Gardner, Scott McCroskey, Snehabala Saravanan, William D. Bradford, Sue L. Jaspersen

**Affiliations:** * Stowers Institute for Medical Research, Kansas City, Missouri,; † Department of Molecular and Integrative Physiology, University of Kansas Medical Center, Kansas City, Kansas

**Keywords:** nuclear envelope, high-throughput screening, membrane proteins, nuclear pore complex, spindle pole body

## Abstract

The nuclear envelope (NE) contains a specialized set of integral membrane proteins that maintain nuclear shape and integrity and influence chromatin organization and gene expression. Advances in proteomics techniques and studies in model organisms have identified hundreds of proteins that localize to the NE. However, the function of many of these proteins at the NE remains unclear, in part due to a lack of understanding of the interactions that these proteins participate in at the NE membrane. To assist in the characterization of NE transmembrane protein interactions we developed an arrayed library of integral and peripheral membrane proteins from the fission yeast *Schizosaccharomyces pombe* for high-throughput screening using the split-ubiquitin based membrane yeast two -hybrid system. We used this approach to characterize protein interactions for three conserved proteins that localize to the inner nuclear membrane: Cut11/Ndc1, Lem2 and Ima1/Samp1/Net5. Additionally, we determined how the interaction network for Cut11 is altered in canonical temperature-sensitive *cut11-ts* mutants. This library and screening approach is readily applicable to characterizing the interactomes of integral membrane proteins localizing to various subcellular compartments.

The nuclear envelope (NE) is a double lipid bilayer that separates the nucleoplasm from the cytoplasm to allow for the compartmentalization of biological processes such as transcription and translation. Both the outer nuclear membrane (ONM) and inner nuclear membrane (INM) are enriched for specific nuclear envelope transmembrane proteins (NETs) that serve a wide variety of functions, including chromatin organization and regulation of gene expression, nuclear shape and dynamics, mechanosensation and signal transduction across the NE (Mekhail and Moazed 2010; Luxton and Starr 2014; Ungricht and Kutay 2015). NETs and their interacting partners at the nuclear periphery have been implicated in numerous human diseases collectively referred to as nuclear envelopathies and laminopathies (Somech *et al.* 2005; Chi *et al.* 2009; Méjat and Misteli 2010; Worman *et al.* 2010; Davidson and Lammerding 2014; Janin *et al.* 2017). Studies of disease-associated NETs in humans have also identified striking patterns of cell and tissue specificity in NET expression and splicing, as well as complex tissue-specific disease pathologies in NET mutants (Korfali *et al.* 2012; Worman and Schirmer 2015). One proposed mechanism to explain these patterns of disease manifestation is that it is the result of disruption of interactions between NETs and their binding partners, which are also expressed in tissue-specific manner (de Las Heras *et al.* 2013). Accordingly, determining the mechanisms behind nuclear envelopathies requires an understanding of both the composition of the NE proteome, as well as the NET interactome.

Despite their clear clinical importance, the identification and functional characterization of NETs and their interacting proteins remains challenging. First, for decades, the list of NETs was small, restricted to a handful of abundant INM proteins. Advances in proteomics in the last two decades has expanded the list of candidate NETs to several hundred (Dreger *et al.* 2001; Schirmer *et al.* 2003; Schirmer and Gerace 2005; Korfali *et al.* 2010; Wilkie *et al.* 2011). However, determining which NETs are enriched at the INM and therefore have the potential to directly interact with the genome and participate in biological processes occurring in the nucleoplasm has traditionally required detailed studies of each NET using electron microscopy (EM). This low-throughput method has been essential to resolve the INM from the ONM, which are only separated by 30-50 nm in most cells. Although super-resolution methods offer an alternative to EM, they too are low -throughput. Smoyer *et al.* developed a high-throughput assay based on split-GFP to screen all known and predicted integral membrane proteins in *Saccharomyces cerevisiae* for access to the INM (Smoyer *et al.* 2016). The list of putative INM components included over 400 proteins, many of which also localize to other subcellular regions. Recent work also suggests that proteins are targeted to the INM for protein degradation through INM-specific quality control pathways (Foresti *et al.* 2014; Khmelinskii *et al.* 2014). This adds yet another layer of complexity to the NET interactome. Additionally, the hydrophobic nature of many NETs makes their isolation and interactome characterization particularly difficult (Pankow *et al.* 2016).

Significant insights into the function of many NETs has emerged from studies in model organisms including the budding yeast *S. cerevisiae* and the fission yeasts *Schizosaccharomyces pombe* and *Schizosaccharomyces japonicus*. For example, genetic and cell biological studies using these systems has identified conserved mechanisms for NETs in NE repair and quality control (Webster *et al.* 2014, 2016; Gu *et al.* 2017; Lee *et al.* 2020), regulation of nuclear size and shape (Neumann and Nurse 2007; Hayles *et al.* 2013; Makarova *et al.* 2016; Cantwell and Nurse 2019) and active lipid metabolism at the INM (Romanauska and Köhler 2018). The founding member of the Sad1-Unc84 (SUN) domain-containing proteins, which play roles in chromosome organization, centrosome function and nuclear migration and positioning in many eukaryotes, was originally identified in a screen for yeast mutants defective in spindle formation (Hagan and Yanagida 1990). Sad1 is a component of the *S. pombe* spindle pole body (SPB), the yeast centrosome-equivalent organelle. Sad1 assists in the insertion of SPBs into the NE to facilitate the nucleation of the mitotic spindle microtubules (Hagan and Yanagida 1995; Bestul *et al.* 2017). In addition, Sad1 also facilitates centromere and telomere tethering in both mitosis and meiosis (Chikashige *et al.* 2006; Swartz *et al.* 2014; Wälde and King 2014; Fernández-Álvarez *et al.* 2016).

Like Sad1, Cut11 is also a component of the SPB involved in SPB insertion (West *et al.* 1998; Bestul *et al.* 2017); however, as the fission yeast ortholog of the conserved integral membrane protein Ndc1 it is perhaps best known for its conserved role in the NE tethering of nuclear pore complexes (NPCs) (Chial *et al.* 1998; Stavru *et al.* 2006). Ima1 is the *S. pombe* ortholog of the mammalian INM protein Samp1/NET5 that has conserved functions in nuclear organization (Hiraoka *et al.* 2011; Steglich *et al.* 2012). It transiently localizes to the SPB early in mitosis in *S. pombe*, similar to its localization to the spindle in mammals (Buch *et al.* 2009). Lem2 is one of the two fission yeast Lap2-Emerin-Man1(LEM) domain-containing proteins (the second being Man1). Unlike Ima1 and Cut11, which transiently localize to the SPB during mitosis, Lem2 is found at the SPB throughout interphase and it is present at the INM during the entire yeast cell cycle (Hiraoka *et al.* 2011). Lem2 and Man1 play at least two roles at the INM: heterochromatin tethering (Steglich *et al.* 2012; Banday *et al.* 2016; Barrales *et al.* 2016; Tange *et al.* 2016; Pieper *et al.* 2020) and regulation of NE composition, integrity and structure (Hiraoka *et al.* 2011; Gonzalez *et al.* 2012; Gu *et al.* 2017; Yang *et al.* 2017; Kume *et al.* 2019; Hirano *et al.* 2020). How these diverse functions of these proteins are controlled is poorly understood in part because we lack sufficient knowledge of the interactome for these NETs, even in model systems such as yeast.

The membrane yeast two-hybrid (MYTH) technology allows for the identification of interactions between full-length integral membrane proteins heterologously expressed in *S. cerevisiae* (Snider *et al.* 2010). Previously, we adapted this technology to study the interactome of Ndc1 in *S. cerevisiae* (Chen *et al.* 2014). Not only did we identify novel Ndc1 interacting proteins, but the MYTH approach enabled us to test *ndc1* mutant alleles for defects in binding to various substrates and better understand the phenotypic differences we observed for these alleles. Here, we have expanded this approach to study fission yeast NE membrane proteins using a newly developed library of 1037 *S. pombe* MYTH prey constructs. Using this library, we performed high-throughput screening to identify interactions for three highly conserved INM proteins of diverse structure and function: Cut11, Lem2 and Ima1. Additionally, we determined how canonical alleles of *cut11* alter the Cut11 interactome to better understand its role at the SPB and NPC in fission yeast. This library is a new resource for the *S. pombe* community to assist in the characterization of integral membrane protein interactions.

## Materials and Methods

### Media preparation and yeast culture

Standard methods were used for both *S. cerevisiae* (Daniel Gietz and Woods 2002) and *S. pombe* (Murray *et al.* 2016) transformation and colony selection. Synthetic drop-out (SD) media lacking the indicated amino acids was prepared by mixing 6.7 g yeast nitrogen base without amino acids with ammonium sulfate, 20 g dextrose (Sigma), 20 g Bacto Agar (VWR) and 0.5-1 g amino acid drop-out powder (Sunrise Scientific) in 1 L of water. Yeast extract with supplements (YES) media was prepared by mixing 5 g yeast extract, 30 g dextrose, 0.2 g each adenine, uracil, histidine, leucine and lysine, in 1 L of water. MYTH screens were performed in 96- or 384-well format on PlusPlates (Singer Instruments). Dilution assays to assess growth for MYTH bait quality control experiments were done using a serial tenfold dilution series spotted onto control (SD-leu-trp) or selection media (SD-leu-trp-ade-his). Growth assays for *cut11-ts* rescue by Pom deletion were done using a fivefold serial dilution series spotted onto YES agar plates incubated at permissive (25°) or restrictive (36°) temperature.

### Generation of MYTH prey library

The coding sequence of each prey of interest was amplified from an *S. pombe* cDNA library (AS One International, Inc.) using KOD Hot Start DNA polymerase (Millipore Sigma), and reactions were cleaned up using the MagSi-DNA Clean paramagnetic beads (Amsbio). The amplicons were cloned into PCR-linearized pPR3-N prey plasmid (Dualsystems Biotech) at the SfiI sites using the NEBuilder HiFi DNA Assembly Kit (New England Biolabs), and were transformed into DH5α competent cells and plated onto 48-well Bioassay Qtrays (Molecular Devices). Automated colony picking was performed using a QPix 420 robotic colony picker (Molecular Devices), followed by high-throughput plasmid prep using a BioMek FXP liquid handling workstation (Beckman Coulter) and Sanger sequencing for insert validation. After sequencing validation, the prey plasmids were transformed into the MYTH prey reporter strain SLJ6830 (NMY61, Dualsystems Biotech) using traditional LiOAc protocol on a Freedom EVO automation platform (Tecan). Transformants were selected on SD-trp media, then picked and arrayed into a final format spanning thirteen 96-well plates using the Tecan EVO. In a similar fashion, MYTH bait coding sequences were gene synthesized (GenScript) and cloned into either the C-terminal (pBT3-STE) or N-terminal (pBT3-N) bait plasmids (pSJ1283 and pSJ1281). The bait plasmids were transformed into SLJ5572 (NMY51; Dualsystems Biotech) and transformants were selected on SD-leu plates. A list of all 1037 prey in the MYTH library is provided in Table S1.

### MYTH screening

Liquid cultures for each bait were spotted onto SD-leu PlusPlates in 96-well format and 10 μl volumes, and incubated for 2 days at 30°. Similarly, the arrayed prey library was spotted in 96-well format on SD-trp PlusPlates. The bait and prey colonies were mated on YPD plates using a RoToR HDA-Robot (Singer Instruments Co. Ltd) and incubated overnight at 30°. Diploids were selected by transferring the resulting colonies to SD-leu-trp media. Following diploid selection, each bait-prey combination was spotted in technical quadruplicate in 384-well format on SD-leu-trp-ade-his PlusPlates supplemented with 25 mM 3-aminotriazole (3-AT) to prevent leaky expression of the *HIS3* reporter gene (Snider *et al.* 2010).

### MYTH analysis

Colony growth was monitored visually, and plates were imaged every 24 hr for 4 days. Colony densities were extracted using a custom FIJI/ImageJ plugin, and downstream analysis was conducted using RStudio (RStudio Team 2020). The code used for density extraction, data analysis and visualization are made available at http://www.stowers.org/research/publications/libpb-1540. Colony growth values obtained using this approach were nearly identical to measurements obtained using other tools designed for analysis of array-based high-throughput screens (Wagih *et al.* 2013; Wagih and Parts 2014) (Figure S1). Variations in diploid colony sizes were accounted for by normalizing test colony densities to diploid colony areas. Diploid colony areas were extracted from SD-leu-trp plate images using standard thresholding techniques and were min-max normalized. The corresponding densities from SD-leu-trp-ade-his + 3-AT plates were then divided by the diploid colony area normalization value. To determine a density cutoff for assigning positive interactions, we manually assigned at least one hundred individual colonies to one of four categories based on strength of interaction: negative, weak, medium or strong (summary statistics for the distribution of densities in each category are listed in Table S2). A density cutoff value equal to the 25^th^ percentile of the “weak” interaction densities was chosen, and this threshold was applied to the entire screen. Interactions were considered “positive” if at least one-half of a prey’s spots on the test plate had a density value greater than the cutoff value. Values shown for positive interactors in Tables S2 and S3 correspond to the averaged density values for all technical replicates of each prey. GO term enrichment analysis was performed for prey that showed a twofold or greater enrichment for a specific bait, using LAGO (Boyle *et al.* 2004), https://go.princeton.edu/cgi-bin/LAGO), using the PomBase GOA annotations for biological processes, a p-value cutoff of 0.01 with Bonferroni correction, and the MYTH prey library as background.

### MYTH confirmation

To confirm MYTH results and eliminate false positives, select bait and prey plasmids were sequentially transformed into the haploid MYTH reporter strain SLJ5572. Following selection on SD-leu-trp, each strain was cultured in SD-leu-trp liquid media overnight at 30°, and a ten-fold dilution series was spotted onto SD-leu-trp and SD-leu-trp-ade-his + 25 mM 3-AT plates incubated at 30° (Figure S3).

### Microscopy and image analysis

Wild-type or *cut11-ts* strains were tagged at their endogenous locus with a C-terminal GFP tag. Cells were cultured in YES media at 25°, and exponentially growing cells were collected and fixed for 20 min with 4% paraformaldehyde as previously described (Bestul *et al.* 2017). Cells were resuspended in 1x PBS and imaged on a Nikon CSU-W1 inverted spinning disk confocal microscope equipped with an Andor EM-CCD camera and an aPlan Apochromat 100x, 1.46 NA oil immersion objective. GFP fluorescence was excited with a 488nm (70mw) diode laser and collected with an ET535/30m emission filter. Data were acquired using Nikon Elements software (Nikon) with z-spacing of 300 nm covering a total volume of 6.3 μm. All strains were imaged on the same day using constant exposure times, laser power and camera gain. Images were processed using FIJI/ImageJ (Schneider *et al.* 2012), National Institutes of Health, Bethesda, MD). To quantify Cut11-GFP levels at the NE, nuclear masks were created by automated thresholding of the Cut11-GFP signal. First, image stacks were maximum projected, background subtracted using a rolling ball radius of 20 pixels and blurred with a Gaussian blur filter. Thresholding was performed using the default algorithm in ImageJ, and nuclei were defined as particles with a size between 4 and 12 μm^2^ and a circularity value of 0.3 or greater. The nuclear mask was then applied to a sum projection of the original image stack to extract mean intensity values for each nucleus. These values were plotted using the GraphPad Prism software package (v 8.4.3).

### Data availability

All strains and plasmids generated for and used in this study will be made available upon reasonable request. Original data and code used for analysis underlying this manuscript are accessible through the Stowers Original Data Repository at http://www.stowers.org/research/publications/libpb-1540. Supplemental material available at figshare: https://doi.org/10.25387/g3.13148642.

## Results

### Generation of an arrayed S. pombe MYTH prey library

**Figure 1:**
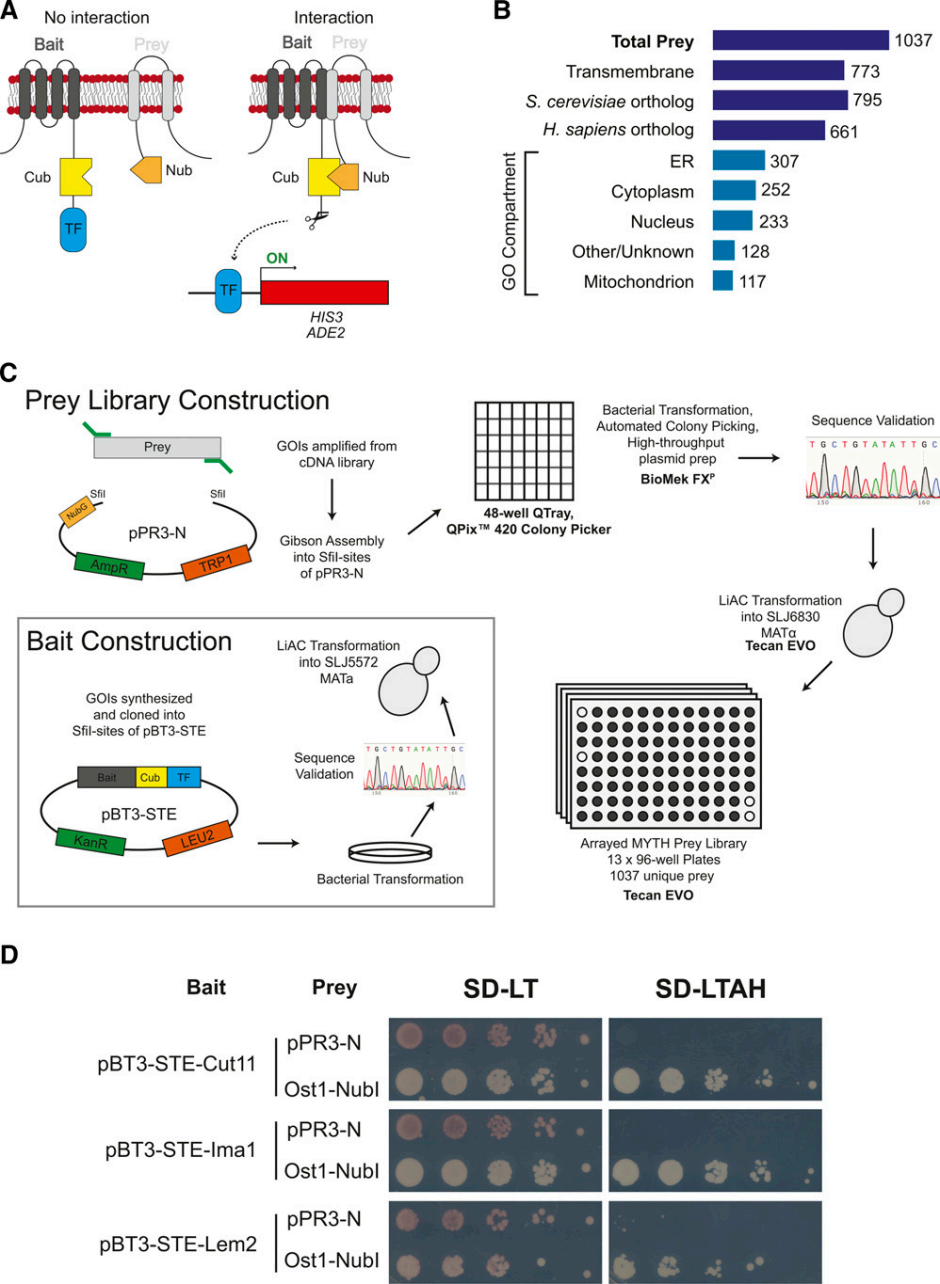
Generation of *S. pombe* MYTH prey library. A) Schematic of split-ubiquitin MYTH approach. Full-length integral membrane bait proteins are fused to the C-terminus of ubiquitin (Cub) and a LexA-VP16 transcription factor reporter (TF). Upon interaction with a prey protein fused to N-terminus of ubiquitin (Nub), the ubiquitin molecule is reconstituted and cleaved by proteases to release TF for expression of *HIS3* and *ADE2* reporter genes. While the schematic depicts a *cis*-interaction between membrane proteins, this system can also detect *trans*-interactions between proteins in membranes of different organelles, or between integral membrane bait and soluble prey, as long as the bait Cub and prey Nub fragments are in the same compartment. B) Diagram of *S. pombe* MYTH prey library composition by protein feature, conservation status and GO Compartment ID. C) Schematic of prey and bait construction approach. See Materials and Methods for detailed description. D) Validation of INM bait proteins for MYTH. Expression of both bait and prey plasmids is confirmed by growth on media lacking leucine (L) and tryptophan (T) (SD-LT). Cell growth on selective media further lacking both adenine (A) and histidine (H) (SD-LTAH) is only observed when each bait is co-expressed with a positive control Ost1-NubI prey protein, but not with the empty prey vector control (pPR3-N).

MYTH was first described by Stagljar *et al.* in 1998 (Stagljar *et al.* 1998) and has been used to identify interactions between integral membrane proteins from multiple species (Miller *et al.* 2005; Paumi *et al.* 2007; Gisler *et al.* 2008; McGee *et al.* 2009; Jin *et al.* 2010). In our system, membrane bait proteins of interest were fused to a C-terminal fragment of ubiquitin (Cub) and a transcription factor (TF) moiety containing both the *E. coli* LexA DNA-binding domain and the herpes simplex virus VP16 activation domain (Figure 1A). Interaction of the bait-Cub fusion protein with prey proteins fused to the N-terminus of ubiqtuitin (Nub) reconstitutes a pseudoubiquitin molecule at the membrane, which is recognized and processed by deubiquitinating proteases, releasing the TF reporter to activate the expression of *HIS3* and *ADE2* reporter genes. This system provides a powerful way to assay for *cis*-interactions between integral membrane proteins in the same membrane (as depicted in Figure 1A) and for interactions between bait and prey membrane proteins present on different membranes that face the same compartment or with soluble prey.

To facilitate high-throughput screening and analysis of integral membrane bait proteins in fission yeast, we generated an arrayed library of *S. pombe* prey proteins fused to Nub (Figure 1A-C, Table S1). We initially targeted ∼1300 proteins including all proteins with one or more known or predicted transmembrane domain. A total of 1037 unique prey constructs were successfully generated, including 81% (773/946) of all integral membrane proteins. The remaining 264 prey include soluble and peripheral membrane proteins with annotated functions at a variety of subcellular locations including the NE, the endoplasmic reticulum (ER) and the mitochondrion (Figure 1B). The arrayed prey library approach allows us to rapidly assess pairwise interactions with hundreds of individual prey proteins in a single assay. Since the position of each prey protein is known, quantification of colony growth and downstream data analysis is streamlined as compared to alternative approaches that require recovery of plasmid DNA and sequencing to identify positive interactions (Snider *et al.* 2010).

### Bait selection, validation and library screening

To demonstrate the utility of the MYTH prey library we next sought to characterize the interactions for a collection of integral membrane proteins known to localize to the INM. Based on their conserved roles at the INM throughout eukaryotes, we focused on the SUN-domain protein Sad1, the nucleoporin (Nup) Cut11, the LEM-domain proteins Lem2 and Man1, and the Samp1/NET5 ortholog Ima1.

Strains expressing the INM baits were tested to confirm that the bait-Cub fusion proteins were expressed and that the growth of these strains on selective media was dependent upon prey interaction. Strains co-expressing individual baits with the empty prey plasmid (pPR3-N) fail to grow on selective media, while co-expression with a positive-control prey containing the Nub fragment that retains its affinity for Cub (Ost1-NubI) reconstitutes the pseudoubiquitin molecule and drives expression of the *HIS3* and *ADE2* reporter genes. Cut11, Ima1 and Lem2 C-terminal bait constructs (pBT3-STE) all passed these initial quality control assays (Figure 1D). Strains expressing Sad1 or Man1 baits were either not expressed or autoactivated (N-Sad1) (Figure S2A). We therefore proceeded with screening Cut11, Ima1 and Lem2 against the prey library. As each localize to distinct subregions of the INM and do not contain similar functional domains, comparison of hits would enable us to identify specific and non-specific interactions. All three baits were screened against the MYTH prey library simultaneously to avoid variability in media composition and screening conditions, allowing for direct comparison of colony size/density across baits.

**Figure 2:**
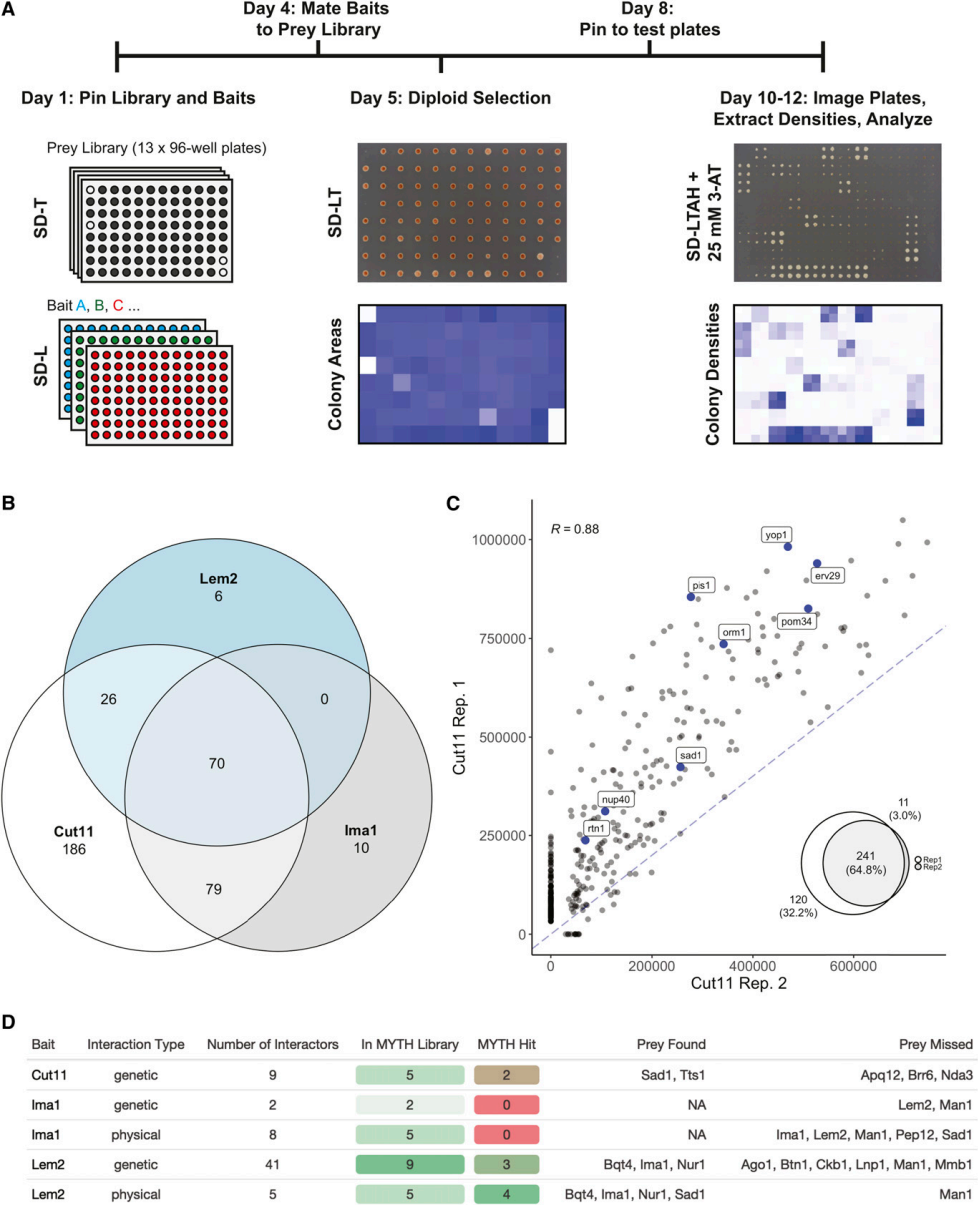
Screening for INM protein interactions using MYTH library. A) Overview of MYTH library screening. Strains expressing the MYTH bait and prey were grown on PlusPlates, mated and diploids were selected by growth on SD-LT media in 96-spot format. Positive bait-prey interactions were identified by monitoring colony growth on selective media (SD-LTAH) supplemented with 3-AT. Colony areas (diploids) or densities (test plates) are extracted and prey identity is assigned based on known prey libray plate layout in an automated fashion. B) Venn diagram illustrating the number of shared or unique interacting prey proteins identified for each bait. C) Comparison of colony densities from two independent Cut11 bait screen replicates. Euler diagram (*inset*) showing the overlap in distributions of interactions identified in each replicate. Proteins known to interact with Ndc1 in *S. cerevisiae* are highlighted and labeled. D) Reported genetic and physical interactors for each bait were examined to determine whether they were present in our prey library and identified as a positive hit in our screens.

To screen the three baits of interest against the MYTH library, strains expressing each bait were crossed with the full MYTH prey library in a high-throughput fashion (Figure 2A). Positive interactions between bait-prey pairs were determined in a semi-automated fashion using a manually defined colony density threshold (*see Materials and Methods*), with larger densities representing stronger interactions between bait and prey pairs (Figure S1A). This approach allowed for the examination of thousands of pair-wise interactions in approximately two weeks from initiation of cultures to completion of analysis.

Of the 1037 unique prey in the library, a total of 377 were identified as hits for at least one of the three INM baits that were screened (Figure 2B, Table S2). We first examined whether our screen identified known physical interactions for each of our baits. Four of the five known Lem2 interacting proteins were identified as Lem2 hits in our screen (Bqt4, Sad1, Nur1, Ima1, Figure 2D). Although no physical interactions have been reported for Cut11 in *S. pombe*, our MYTH screen identified 75% (9/12) of the prey that are orthologs of proteins reported to physically interact with Ndc1 in *S. cerevisiae* and were in our prey library (Figure 2C). In contrast, our screen failed to detect any of the five reported physical interactors for Ima1 that were present in our library. We noted that Man1 was not functional as a bait or prey, as the prey was not found to interact with Lem2 or Ima1, despite previously being identified as an interactor for both (Hiraoka *et al.* 2011). We were unable to generate a functional Sad1 bait, however, the Sad1 prey construct was functional and recapitulated the known interaction with Lem2 (Hiraoka *et al.* 2011; Vo *et al.* 2016). Comparison of two independent screens using the same bait (Cut11) showed a high level of reproducibility in both the identity and strength of the prey interactions (241/372, 65% shared hits; Pearson’s correlation = 0.88) (Figure 2C). Variability between replicates was most prominent for weak interactions, which may be attributable to variation in media across experiments. Together, these results demonstrate that our MYTH screen identified many known interactions with a high level of reproducibility; however, some proteins are incompatible with the MYTH system. This is likely due to defects in protein stability or folding, localization, or membrane topology introduced by expression as a bait or prey fusion protein.

MYTH and other high-throughput screening techniques are powerful discovery tools, but one limitation for these approaches is the presence of false positive and false negative hits present in these datasets. Our screens were performed in the presence of the His3 competitive inhibitor 3-AT to increase stringency and limit the number of false positive hits identified. However, other factors including differences in mating efficiencies and slight variations in diploid colony densities, which are difficult to control for in high-throughput format, could also influence our screening results. To examine these possibilities we conducted traditional spot growth assays for a select subset of bait-prey combinations in which both bait and prey plasmids were expressed in a haploid background (Figure S3). These experiments largely agreed with the results obtained from the high-throughput screen with respect to strengths of interactions and bait specificity. However, there were some differences (*i.e.*, Hut1, Bem46, Vps55), suggesting that variables including mating efficiencies and/or cell densities could introduce errors during screening and highlighting the importance of additional validation of MYTH hits prior to follow-up studies.

### Comparison of interactions identified for each bait

**Figure 3:**
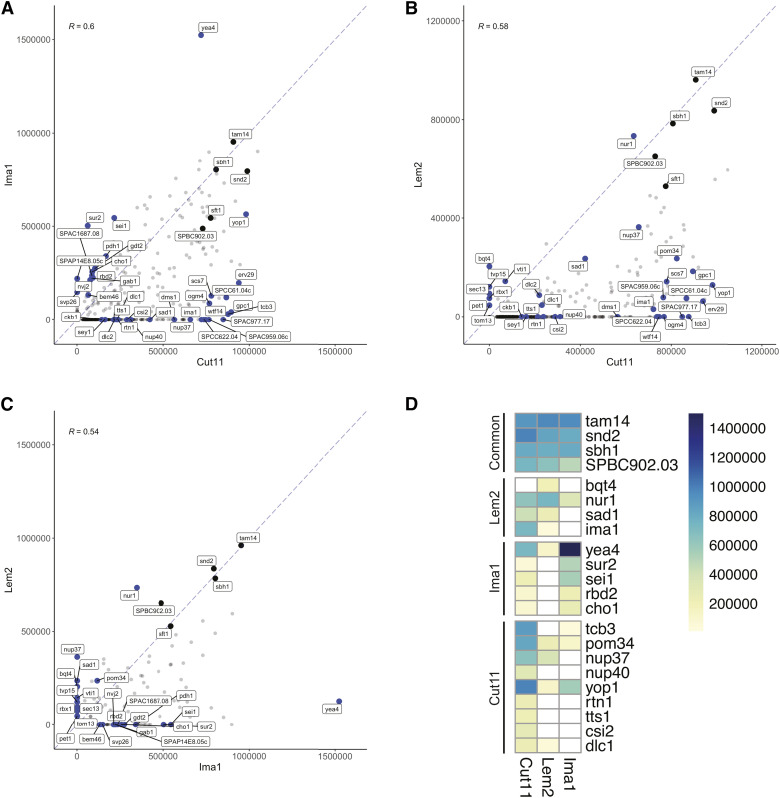
Comparison of INM bait interactomes. A-C) Scatter plots showing pairwise comparisons of average colony density values for prey identified as positive interactors for at least one bait protein in our screen. Prey that have a similar strength of interaction with both baits align on the diagonal dashed line. Prey of interest for each bait are annotated in blue, while four prey that were among the top interactions for all three baits are annotated in black. Pearson correlation coefficients (R) are listed for each comparison. D) Heat map representation of select prey of interest that are discussed in text. Prey that showed no interaction with each bait are shown in white.

We next performed pairwise comparisons between each bait to identify interactions that were common and those that were unique or enriched for specific baits. The strong positive correlation observed between replicates was not observed in comparisons between baits, and only 70 prey (18.5% of all hits) displayed interactions with all three baits (Figure 2B; Figure 3). A subset of these common interactors were among the strongest interactions identified for all three baits and likely represent non-specific interactions resulting in false positives. However, this group also included prey that displayed significant preferences for certain baits. For example, the transmembrane Nup Pom34, which forms a complex with Ndc1 in *S. cerevisiae* (Onischenko *et al.* 2009), interacted with all three baits yet showed a 5-7-times stronger interaction with Cut11 compared to Lem2 and Ima1 (Figure 3D). GO term enrichment analysis of the hits that were uniquely enriched (> twofold increased over other baits) for each bait failed to identify any functional enrichment for the interacting prey. We therefore focused our analysis on prey that shared localization or predicted function with each bait, as well as those interactions that were unique or significantly enriched. Interactions of interest for each bait are discussed below.

#### Lem2 MYTH interactors:

Lem2 is a conserved integral membrane protein that serves multiple functions at the INM. A total of 82 prey interacted with Lem2, only 6 of which were unique interactions (Bqt4, Sec13, Tvp15, Rbx1, Pet1, Tom13). We observed a strong unique interaction with the INM protein Bqt4, which anchors telomeres to the NE and binds directly to Lem2 to retain it at the NE (Hirano *et al.* 2018). The other unique interactions were very weak, but Tvp15 was confirmed as an interactor in follow-up studies where plasmids were directly expressed in the same cell (Figure S3). Although not unique for Lem2, the Lem2-interacting protein Nur1 (Mekhail *et al.* 2008; Banday *et al.* 2016; Iglesias *et al.* 2020) was identified as the second strongest Lem2 interactor and was enriched threefold relative to Cut11 and Ima1. Identification of Bqt4 and Nur1 is consistent with the role of Lem2, Bqt4 and Nur1 in silencing and retention of centromeres and telomeres at the nuclear periphery (Gonzalez *et al.* 2012; Banday *et al.* 2016). Lem2 interacts directly with Bqt4 and Nur1 through its N-terminal LEM domain (Barrales *et al.* 2016), providing evidence that MYTH is able to identify *bona fide* binding proteins for NETs.

We further examined Lem2 hits in an attempt to gain insight into proteins that may assist in its functions in heterochromatic gene silencing, telomere positioning and centromere tethering at the SPB. Unfortunately, many of the heterochromatin-associated factors that showed genetic interactions with Lem2 (Barrales *et al.* 2016) were absent from our prey library, and none of the 14 heterochromatin factors present in our library interacted with Lem2 (Ago1, Stc1, Raf2, Pcu4, Chp1, Swi6, Clr4, Lem2, Man1, Nup85, Mmi1, Red1, Shf1, Brl1). Of the factors associated with telomere localization present in our library, Sad1, Bqt3 and Bqt4 were found to interact with Lem2. The only strong interaction we observed with known SPB-localized proteins was with Sad1, which has previously been shown to interact with Lem2 (Hiraoka *et al.* 2011; Vo *et al.* 2016). Thus, despite confirming known interactions with Sad1, Bqt4 and Nur1, interactions that may physically connect Lem2 with components of the heterochromatin machinery still remain elusive (Braun and Barrales 2016).

**Figure 4:**
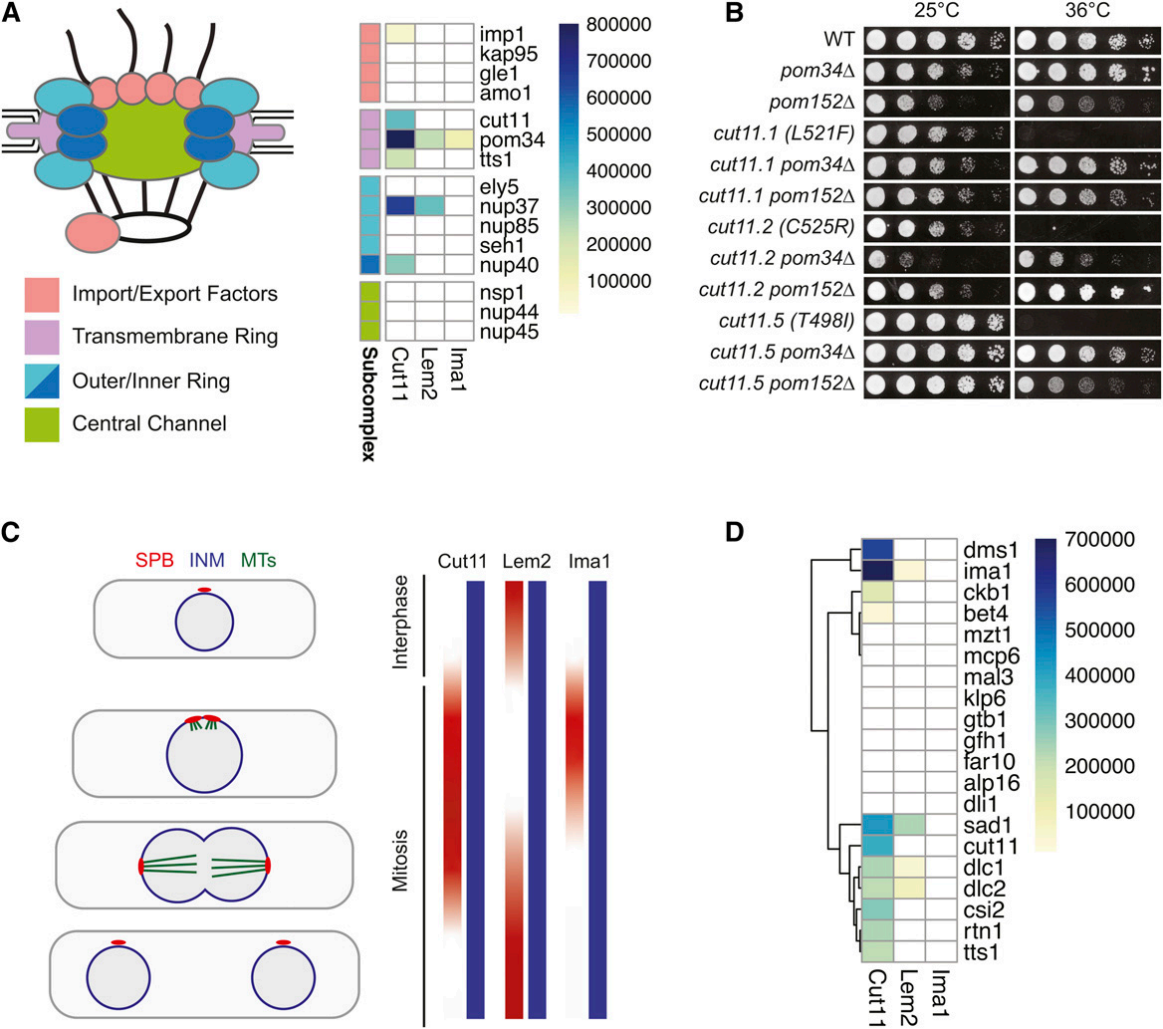
Interactions identified with NPC and SPB components. A) Cartoon representation of NPC structure (*left*) with subcomplexes colored to match groupings shown in heat map of prey that are components of the NPC (GO: 0005643) (*right*). B) Dilution assays assessing growth of *cut11* wild-type or temperature-sensitive alleles at permissive (25°) and restrictive (36°) temperatures. Deletion of either Pom34 or Pom152 rescues the temperature sensitivity of all *cut11-ts* alleles. C) Cut11, Lem2 and Ima1 each show enrichment at the SPB but at different stages of the cell cycle. Lem2 localizes to the SPB throughout interphase but is absent during early stages of mitosis. In contrast, both Cut11 and Ima1 localize to the SPB during mitotic entry during the period where Lem2 is absent. All three proteins also localize throughout the INM during the entire cell cycle. D) Heat map of interactions with prey proteins that are components of the SPB (GO:0005816). Dendrogram represents row-wise hierarchical clustering generated using default parameters of the ‘pheatmap’ package in R.

Lastly, we examined the Lem2 interacting prey for factors potentially associated with NPC quality control at the NE. In *S. pombe*, Lem2 is required for maintaining NE morphology and regulating membrane flow from the ER into the NE to control nuclear size (Gonzalez *et al.* 2012; Kume *et al.* 2019; Hirano *et al.* 2020). However, a direct role for Lem2 in NPC quality control has not been shown in S. *pombe*, and Lem2 is not known to interact directly with any components of the NPC or the ESCRT (endosomal sorting complexes required for transport) machinery that facilitate NE/NPC quality control in other organisms. In budding yeast, the Lem2 ortholog Heh1/Src1 binds with luminal domains of the transmembrane Nup Pom152 and displays genetic interactions with Nups in multiple subcomplexes (Yewdell *et al.* 2011). This interaction with the NPC is thought to allow Heh1/Lem2 to recruit the ESCRT component Chm7 to regions of the NE that contain defects in NPC assembly to ensure NE compartmentalization is maintained (Webster *et al.* 2014, 2016). Although we were unable to generate a functional *S. pombe* Pom152 MTYH prey, Lem2 interacted with the transmembrane Nup Pom34 (Figure 4D). We also observed an interaction between Lem2 and the inner ring component Nup37, which was recently found to associate with Lem2 in mammalian cells (Moser *et al.* 2020). Our results, in combination with the genetic interaction between Lem2 and Chm7 in *S. pombe* (Gu *et al.* 2017), their physical interaction in *S. japonicus* (Pieper *et al.* 2020) and genetic interactions between Nups and the ESCRT machinery (Frost *et al.* 2012) suggests that a similar mechanism could be conserved.

#### Ima1 MYTH interactors:

Ima1 is the fission yeast ortholog of the mammalian INM protein Samp1/NET5 (Schirmer *et al.* 2003; Buch *et al.* 2009), for which there is no apparent ortholog in *S. cerevisiae*. Samp1 influences the distribution of other INM proteins, including the Sad1 ortholog Sun1, is required for centrosome positioning and tethering to the NE during interphase, and localizes to transmembrane actin-associated nuclear (TAN) lines where it stabilizes SUN-domain containing linker of nucleoskeleton and cytoskeleton (LINC) complexes to promote nuclear movement (Buch *et al.* 2009; Gudise *et al.* 2011; Borrego-Pinto *et al.* 2012). In *S. pombe*, Ima1 localizes to distinct sub-regions of the NE where it interacts with specific heterochromatic regions of the genome (Steglich *et al.* 2012). Despite enrichment of Ima1 near the central core regions of the centromeres, Ima1 is not required for centromere tethering at the SPB (Hiraoka *et al.* 2011; Hou *et al.* 2013). The function of Ima1 in *S. pombe* is unclear, though it appears to share a redundant role in nuclear membrane morphology and structure with the LEM-domain proteins Lem2 and Man1 (Hiraoka *et al.* 2011). Therefore, identification of Ima1 interactors, particularly novel binding partners, would shed light on its function and guide future functional studies.

Ima1 has eight reported physical interactions, five of which are present in the MYTH prey library (Ima1, Lem2, Man1, Pep12, Sad1). Unfortunately, none of these interactors were identified as Ima1 hits in our screen. While this could be due to expression or functionality of either the bait or prey constructs, the fact that we identified the Ima1 and Sad1 prey constructs in our Cut11 and/or Lem2 screen makes this unlikely. It is possible that Ima1 interactions with these proteins may be regulated in a manner not recapitulated in the heterologous system.

In both fission yeast and mammals, Ima1/Samp1 displays cell cycle dependent changes in its localization from the INM to the SPB/centrosome-associated membrane. Given this conserved, transient recruitment of Samp1/Ima1 to the centrosome during mitosis, we next examined the list of Ima1 interactors for factors known to localize to the SPB. Although we did not observe any interactions between the Ima1 bait and SPB-localized prey, we noted that Ima1 prey showed a specific and strong interaction with the Cut11 bait (Figure 4D). Ima1 localizes to the SPB specifically during the early stages of mitosis, temporally overlapping with the kinetics of Cut11 recruitment to the SPB during SPB insertion (Figure 4C) (West *et al.* 1998; Hiraoka *et al.* 2011). It is thus possible that Ima1 localization to the SPB is facilitated by its interaction with Cut11, although its interaction with Sad1 may also contribute.

We next examined the twelve interactors that were uniquely identified for Ima1. The strongest of these interactions was with Sur2, a conserved sphingolipid metabolic enzyme with bi-functionality as a sphingosine hydroxylase and ∆4-desaturase (Haak *et al.* 1997; Sperling *et al.* 2001; Nakase *et al.* 2010; Vacchina *et al.* 2012). While a functional role for Sur2 at the INM remains to be explored in fission yeast, Sur2 was found to have access to the INM in *S. cerevisiae* (Smoyer *et al.* 2016), and the mammalian sphingolipid hydroxylase Smpd4 is enriched at the NE and physically interacts with components of the NPC (Cheng *et al.* 2019; Magini *et al.* 2019). Further, Sur2 substrates, including the long-chain base dihydrosphingosine, are enriched at the nuclear membrane where they play a key role in maintaining nuclear morphology in both yeast and mammalian cells (Hwang *et al.* 2019). This suggests that an interaction with Ima1 may retain a pool of Sur2 at the INM in *S. pombe* where it could influence properties of the NE membrane by controlling local sphingolipid content.

Ima1-enriched prey included other factors implicated in lipid biogenesis and membrane organization. For example, Ima1 had a strong interaction with the seipin ortholog Sei1, which has a conserved role in lipid droplet biogenesis at the ER and stabilizes lipid droplet-ER contact sites (Grippa *et al.* 2015). Recently, Sei1 was found to localize to the INM in budding yeast where it is required for the formation of membrane bridges between the INM and nuclear lipid droplets (Romanauska and Köhler 2018). We also observed an interaction with Nvj2, a lipid-binding protein that localizes to membrane contact sites between the nucleus and vacuole and forms membrane bridges between the ER and Golgi network to alleviate high levels of ceramides through nonvesicular transport (Toulmay and Prinz 2012; Liu *et al.* 2017). Although this function in ceramide transport was not attributable to the perinuclear ER pool of Nvj2, it is possible that similar mechanisms exist to transport ceramides out of the NE, and its interaction with Ima1 presents additional evidence linking Ima1 with factors that may influence membrane composition at the INM. Both Sei1 and Nvj2 interactions were further confirmed by retransformation and dilution of haploid cells, making them ideal candidates to be to be further pursued (Figure S3). Other Ima1 interactors implicated in lipid metabolism include the rhomboid protease Rbd2, which cleaves and activates the sterol regulatory element-binding protein (SREBP) transcription factor Sre1 and is present at the INM in *S. cerevisiae* (Kim *et al.* 2015; Hwang *et al.* 2016; Smoyer *et al.* 2016) and the phosphatidylcholine synthesis protein Cho1 (Kanipes *et al.* 1998). The remainder of the Ima1-enriched interacting prey are largely uncharacterized proteins with a variety of predicted functions including ER-associated protein modifications and vesicular transport, three of which (Yea4, Bem46, SPAP14D8.05c) physically interact with the INM protein Man1 (Vo *et al.* 2016). As many of these factors are conserved in humans, further characterization of Ima1 and its interacting proteins in fission yeast will provide insight into their potential conserved functions at the nuclear envelope.

#### Cut11 MYTH interactors:

Cut11/Ndc1 is a transmembrane Nup conserved between yeast and vertebrates and is required for NPC assembly (Mansfeld *et al.* 2006; Madrid *et al.* 2006; Stavru *et al.* 2006). In budding yeast Ndc1 has genetic and physical interactions with the transmembrane Nups Pom34 and Pom152 (Onischenko *et al.* 2009; Chen *et al.* 2014). These interactions are required for NPC assembly and influence the distribution of Ndc1 in the NE by competing with the SUN-domain protein Mps3 for a shared binding site on Ndc1 (Chen *et al.* 2014). We previously used the MYTH system to show that deletion of Pom152 rescues *ndc1* temperature sensitive (ts) alleles by increasing Ndc1-Mps3 interactions to promote Ndc1 localization at the SPB (Chen *et al.* 2014). Although we were unable to generate a functional *S. pombe* Pom152 prey construct, we observed a strong interaction between Cut11 and Pom34 from our MYTH screen (Figure 4A). Further, deletion of either Pom34 or Pom152 rescued growth of each of the three causative mutations identified in *cut11-ts* strains (L521F (*cut11.1*), C525R (*cut11.2/3/4*) and T498I (*cut11.5/6*), Figure 4B) (Zhang and Oliferenko 2014). The conservation of physical and genetic interactions between Ndc1/Cut11 and the Poms suggests that similar mechanisms controlling Cut11 localization and function in the NE may be conserved in fission yeast.

In addition to the Poms, Ndc1 also interacts with the structural nucleoporins Nup53 and Nup59 and the ER membrane-bending proteins Rtn1 and Yop1 to induce and stabilize membrane curvature that occurs during NPC assembly (Dawson *et al.* 2009; Onischenko *et al.* 2009; Casey *et al.* 2012, 2015). Similar interactions between Ndc1 and Nup53 occur in *Xenopus* (Hawryluk-Gara *et al.* 2008) and vertebrate systems (Mansfeld *et al.* 2006). Many of these factors were present in our prey library and were identified as strong, often unique, interactors for Cut11, including the *S. pombe* Nup53/59 ortholog Nup40, Rtn1, and Yop1 (Figure 3, Figure 4A). Additionally, both Yop1 interacting proteins, Yip1 (SPCC61.04c) and Sey1, which work together to form highly curved ER membrane tubules (Voeltz *et al.* 2006; Hu *et al.* 2008, 2009), facilitate lipid transfer between membranes and organelles (Voss *et al.* 2012) and influence NPC organization (Casey *et al.* 2015) interacted with Cut11 (Figure 3A). Cut11 interacted with a specific subset of NPC components, as many Nups in our prey library showed no interaction with Cut11, including the structural Nups Ely5 and Nup85, and the FG-Nups Nsp1, Nup44 and Nup45 (Figure 4A). We did observe an interaction between Cut11 and Nup37, a structural Nup that is conserved in vertebrates but missing in *S. cerevisiae*. This suggests that a direct interaction between Cut11 and Nup37 may help to anchor the Nup107-160 subcomplex, the major structural component of the NPC, to the pore membrane in *S. pombe*. A direct Cut11-Nup37 interaction supports previous studies that identified genetic interactions between Nup37 and Ndc1 in *Aspergillus nidulans* (Liu *et al.* 2009) and is consistent with the proposal that Nup37 serves as a tether connecting the NPC scaffold and the transmembrane Nups in *S. pombe* (Bilokapic and Schwartz 2012). This interaction may also promote recruitment of the Nup107-160 complex to sites of NPC assembly, similar to the POM121-mediated recruitment reported in metazoans (Doucet and Hetzer 2010). Our screen also revealed an interaction between Cut11 and Tts1, a conserved reticulon-binding protein that has functions in membrane-shaping at the ER and NE (Chadrin *et al.* 2010; Zhang and Oliferenko 2014 p. 201; Urade *et al.* 2014). This was particularly exciting, as Tts1 displays genetic interactions with Cut11 in *S. pombe*, is implicated in NE remodeling during SPB insertion and in controlling NPC distribution during mitotic NE expansion (Zhang and Oliferenko 2014) and co-purifies with fission yeast NPCs (Iglesias *et al.* 2020). (Iglesias *et al.* 2020). Moreover, the interaction with Tts1 is reproducable and specific to Cut11 (Figure S3).

In addition to its function at the NPC, Cut11 is also required for SPB insertion and tethering within the NE during mitosis. In budding yeast, the SUN-domain protein Mps3 controls the distribution of the Cut11 ortholog Ndc1 in the NE and facilitates its recruitment to the SPB, where it forms a membrane ring structure alongside other members of the SPB Insertion Network (SPIN) (Jaspersen and Ghosh 2012; Kupke *et al.* 2017; Chen *et al.* 2019). We have recently found that Cut11 forms similar ring-like structures during SPB duplication and insertion in *S. pombe* (unpublished data); however, the mechanisms that regulate Cut11 distribution and localization at the SPB remain unknown. To identify potential Cut11 interactors at the SPB, we examined interactions with known SPB-associated proteins (Figure 4D). We observed a strong interaction with the Mps3 ortholog Sad1, providing the first evidence for a direct interaction between these two proteins in *S. pombe* and suggesting that Sad1 may similarly promote Cut11’s localization at the SPB.

Cut11 also interacted with Csi2, which localizes to the SPB and is implicated in bipolar spindle formation, microtubule dynamics and chromosome segregation (Costa *et al.* 2014). Also identified was Ckb1, the beta-subunit of casein kinase CK2, which phosphorylates the HP1 protein Swi6 to promote heterochromatic silencing at centromeres (Roussou and Draetta 1994; Shimada *et al.* 2009). Interestingly, we also identified SPB components with meiosis-specific functions. For example, Cut11 interacted strongly with Dms1, which localizes to the SPB specifically during meiosis II and recruits Spo15 for meiotic SPB remodeling to ensure proper SPB number and function (Ikemoto *et al.* 2000; Ohta *et al.* 2012; Blyth *et al.* 2018). Cut11 also interacted with the dynein light-chain proteins Dlc1 and Dlc2. Dlc1 localizes to the SPB throughout mitosis and meiosis and was shown to interact with the meiosis-specific SPB component Kms1 by traditional yeast two-hybrid screen (Miki *et al.* 2004). Dynein motors are required for the “horse-tail” movements of telomere-associated SPBs during meiosis (Yamamoto *et al.* 1999; Miki *et al.* 2002; Niccoli *et al.* 2004; Tomita and Cooper 2007) and promote chromosome segregation during mitosis (Courtheoux *et al.* 2007) and meiosis (Davis and Smith 2005; Chacón *et al.* 2016). Together, these findings identify multiple putative new interacting proteins for Cut11, including components of the NPC and SPB, and demonstrate that the use of a heterologous system for the MYTH screening allows for the identification of interactions that may occur throughout the life cycle.

### Application of MYTH to determine how mutations alter interaction networks

**Figure 5:**
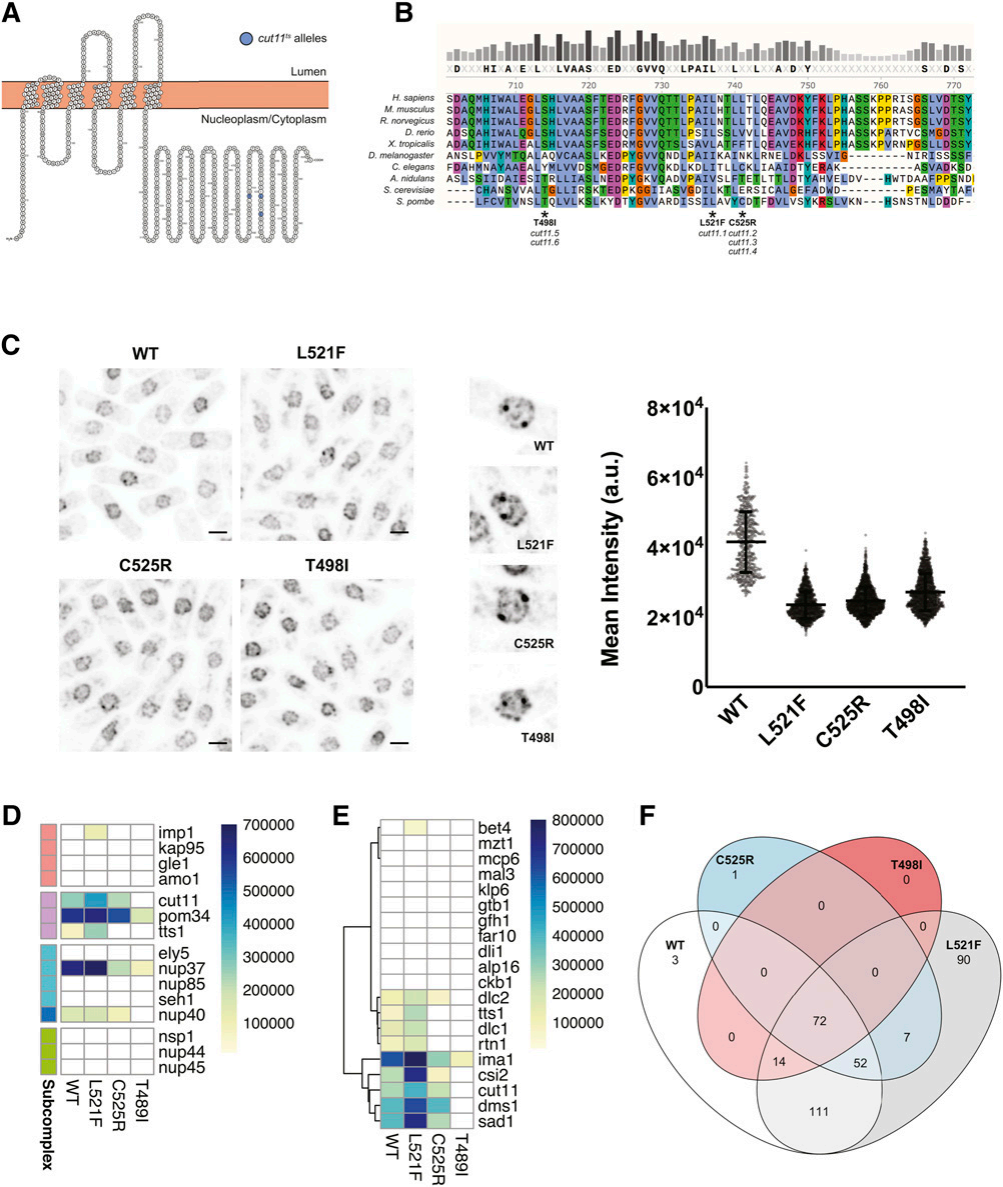
Effect of *cut11* mutations on the Cut11 interactome. A) Schematic of Cut11 topology in the NE, with mutations identified in *cut11-ts* alleles highlighted. All three mutations cluster in a similar region of the C-terminal portion of Cut11 that faces the nucleoplasm/cytoplasm (image generated using Protter, http://wlab.ethz.ch/protter/start/). B) C-terminal regions of Ndc1/Cut11 from multiple species aligned using ClustalOmega in SnapGene (v 5.1.4.1), with coloring based on amino acid properties and conservation. The causative mutation of each *cut11-ts* allele is annotated below the alignment. C) (*left*) Representative images of Cut11 wild-type or mutant GFP fusion proteins, scale bar is 3 microns. (*center*) Images of individual mitotic nuclei showing recruitment of Cut11-GFP to the two SPBs (images show a 7.5x7.5 micrometer field of view). (*right*) Quantification of mean Cut11-GFP intensity values for individual nuclei in wild-type or mutant Cut11 strains. D-E) Heat maps of interactions between Cut11 baits and NPC (D) or SPB (E) components. The subcomplex color scheme in (D) is the same as used in Figure 4A. Dendrograms represent row-wise hierarchical clustering generated using default parameters of the ‘pheatmap’ package in R. F) Venn diagram illustrating the overlap in the interacting preys identified for each Cut11 bait protein.

We previously used the MYTH system to identify proteins that interact with Ndc1 at the SPB and NPC and to characterize how these interactions are modulated in *ndc1* mutants (Chen *et al.* 2014). As we observed conservation of physical and genetic interactions with Cut11 and the orthologous NPC and SPB components in fission yeast (Figure 4), we next examined the effects of canonical *cut11-ts* alleles on Cut11 localization and interactions. The original *cut11.1* mutant allele, leucine 521 to phenylalanine (L521F), is defective in SPB insertion and bipolar spindle formation, but has not been associated with defects in NPC assembly (West *et al.* 1998). Interestingly, this allele was not suppressed by increased levels of Tts1, while all other known alleles were, including *cut11.2/3/4*, which contain a mutation of cysteine 525 to arginine (C525R), and *cut11.5/6* that contain a mutation of threonine 498 to isoleucine (T498I) (Zhang and Oliferenko 2014). All three residues map to the C-terminal tail of Cut11 that faces the nucleoplasm/cytoplasm, with L521F being highly conserved (Figure 5A-B). Visualization of each of the mutant proteins C-terminally tagged with GFP showed that all were expressed at similar levels and localized to punctate structures throughout the NE in interphase and to two bright foci in dividing cells (Figure 5C). This suggests that although these mutants show decreased protein levels relative to wild-type even at the permissive temperature (25°), they properly localize to both NPCs and SPBs similarly to wild-type Cut11.

To begin to understand the phenotypic differences reported for the *cut11-ts* mutants, we introduced the corresponding L521F, C525R or T498I mutations into the Cut11 MYTH bait and validated their expression and functionality (Figure S2B). The mutants were screened against the MYTH prey library simultaneously with wild-type Cut11, allowing for direct comparison of colony size/density across baits. Interestingly, the interactome of *cut11.1* (L521F) was largely similar to that of wild-type Cut11, with strong interactions with NPC components (Nup37, Pom34, Nup40, Cut11) and SPB components (Sad1, Ima1, Csi2) (Figure 5D-F). In contrast, both C525R and T498I mutants showed global alterations to their interactomes including reduced affinity for both NPC and SPB components (Table S3). Inspection of the interaction with Tts1 showed an increase in binding for L521F compared to wild-type and a complete loss of binding for both C525R/T498I. Thus, genetic suppression of *cut11* alleles by Tts1 overexpression correlates with differences in the ability of the Cut11 mutant proteins to interact with Tts1. These results demonstrate that MYTH is a simple alternative tool to study the effect of mutations on protein interactions to provide additional insight into phenotypes observed in *S. pombe*.

## Discussion

Proteins that localize to the NE play critical roles in nuclear and chromatin organization, regulation of gene expression, lipid biosynthesis and membrane structure. To assist in the functional characterization of integral membrane proteins that localize to the NE, we developed an arrayed membrane yeast two-hybrid prey library. Screening of three known INM proteins against this library confirmed many previously reported interactions and identified novel interactions that are intriguing candidates for further mechanistic studies. It is important to note that this screen is performed in a heterologous system, and although we have previously observed MYTH baits properly localizing to the NE in budding yeast (Chen *et al.* 2014), the specific subcellular location where the interactions are occurring in this screen has not been examined. Thus, for follow up studies it will be important to confirm where these interactions take place using orthogonal approaches in their *in vivo* setting in *S. pombe*. Additionally, although our focus was on INM proteins, this library and screening approach is not specific to the NE. Rather, MYTH is immediately applicable to survey interactions that occur at other membranes and organelles in either a high-throughput or targeted approach.

The MYTH system is also a valuable tool for determining how mutations of integral membrane proteins affect their protein interactions. By combining MYTH with genetic approaches available in *S. cerevisiae* and *S. pombe*, we have identified conserved interactions between Ndc1/Cut11 and components of the NPC and SPB that control its distribution in the NE. Characterization of the interaction profiles for canonical *cut11-ts* alleles identified changes in interactions with putative Cut11 binding partners at the SPB and NPC. These studies also revealed a direct physical interaction between Cut11 and the conserved membrane protein Tts1.

The observation that *cut11-ts* alleles differentially alter interactions with Tts1 provides valuable new insight into the mechanisms by which Tts1 and Cut11 may work together at the NE. In addition to its role in controlling NPC distribution during NE expansion, Tts1 deletion also exacerbated the spindle defects observed in *cut11-ts* mutants. The mechanism by which Tts1 exerts its function at the SPB remained unclear, as Tts1 does not localize to the SPB and is not required for localization of Cut11 to the SPB (Zhang and Oliferenko 2014). It was therefore proposed that Tts1 likely promotes NE remodeling during SPB insertion by regulating membrane lipid composition or dynamics. Our data showing that mutants that do not bind to Tts1 (C525R and T498I) still localize to the SPB (Figure 5C) provides additional evidence that binding to Tts1 is not required for recruitment of Cut11 to the SPB or for SPB insertion.

Our results also allow us to address the curious observation that Tts1 overexpression rescues all *cut11-ts* alleles except for *cut11.1* (Zhang and Oliferenko 2014). Our MYTH data show that the *cut11.1* L521F mutation increases binding between Cut11 and Tts1, while both C525R and T498I mutations prevent binding to Tts1. The correlation between the genetic and physical interactions between Tts1 and Cut11 mutants could be explained by at least two potential mechanisms. As Tts1 localizes to the NE/ER and NPC but not the SPB (Zhang and Oliferenko 2014), increased levels of Tts1 could act as a sink to retain Cut11 at these locations at the NE, potentially reducing the amount of Cut11 able to localize to the SPB to facilitate insertion. In this scenario, the impact of Tts1 overexpression is dependent upon its ability to bind to Cut11, and therefore is not observed in C525R or T498I mutants. A mechanism by which Tts1 overexpression competes with SPB components for Cut11 binding is analogous to our data from budding yeast and fission yeast showing that *ndc1/cut11-ts* alleles can be rescued by reducing affinity for the NPC by disrupting interactions with the Pom nucleoporins (Chen *et al.* 2014) (Figure 4B). Alternatively, Tts1 overexpression may rescue *cut11-ts* mutants through an indirect mechanism, potentially restoring some of the global changes to the Cut11 interactome seen in C525R and T498I mutants in ways that are not recapitulated in the L521F mutant. While the correlation between the genetic and physical interactions between Tts1 and Cut11 mutants is intriguing, more work is required to determine which of the potential mechanisms is occurring.

In budding yeast, distribution of Ndc1 between the NPC and SPB is facilitated by the SUN protein Mps3. Although we observe a conserved interaction with the *S. pombe* SUN protein Sad1, it is unclear whether Sad1 serves a similar function given that its localization at the INM is restrained to the regions near the SPB. It is likely that Sad1 serves a more passive role in *S. pombe*, acting as an anchor to retain Cut11 at the SPB but not actively shuttling Cut11 in the NE. In this scenario, recruitment of Cut11 to the SPB during mitosis may be controlled by altering the affinity for Cut11’s binding partners at the NPC or NE. Our data shows that changes within the C-terminus of Cut11 influence its protein interactions. Multiple residues in this region are phosphorylated in a cell-cycle dependent manner (Koch *et al.* 2011; Swaffer *et al.* 2018) and could drive similar alterations to the Cut11 interactome to promote its recruitment to the SPB.

It also remains unclear how the C525R and T498I mutant proteins localize to the SPB, as many of the SPB interactions including Sad1 are significantly reduced or completely lost in these mutants. Both C525R and T498I are able to bind to Ima1, which localizes to the mitotic SPB with similar kinetics as Cut11 (Figure 4C). However, a direct role for Ima1 in Cut11 recruitment has not yet been reported. It is also likely that other SPB components that are not in our MYTH prey library, such as the KASH-protein Kms2 and the mitotic regulator Cut12, remain capable of binding and recruiting C525R and T498I mutant Cut11 proteins. The interactome data for the *cut11* alleles presented in this study will help guide future mechanistic studies exploring Cut11 function. MYTH may also be a helpful tool to identify mutations that will allow for separation of SPB and NPC function, which would be useful for studies characterizing the role of this conserved nucleoporin in NPC assembly and insertion into the NE.
